# Self-perceived stress is associated with adiposity and atherosclerosis. The GEA Study

**DOI:** 10.1186/s12889-015-2112-8

**Published:** 2015-08-14

**Authors:** Janinne Ortega-Montiel, Carlos Posadas-Romero, Wendy Ocampo-Arcos, Aida Medina-Urrutia, Guillermo Cardoso-Saldaña, Esteban Jorge-Galarza, Rosalinda Posadas-Sánchez

**Affiliations:** Department of Endocrinology, National Institute of Cardiology “Ignacio Chavez”, Juan Badiano No. 1 Col. Seccion XVI, Tlalpan, 14080 Mexico City, Mexico

## Abstract

**Background:**

A growing body of evidence suggests that psychological stress is an independent cardiovascular risk factor. Obesity prevalence shows accelerating trends worldwide, and is known to be associated with a range of comorbidities and survival. The aim of this study was to assess the relationship between self-perceived psychological stress with parameters of adiposity, metabolic syndrome, and subclinical atherosclerosis in Mexican participants.

**Methods:**

Metabolic Syndrome was defined using the Adult Treatment Panel III criteria, obesity was defined as BMI >30, subclinical atherosclerosis disease was determined by computed tomography, and carotid intima media thickness was determined by ultrasonography. Self-perceived psychological stress was assessed using a single-item questionnaire.

**Results:**

A total of 1243 control subjects were included in the sample, mean age 54.2 ± 9 years old; the prevalence of chronic self-perceived psychological stress (>5 years) was 10.13 %, female gender (62.7 %), obesity prevalence (48.4 %), and self-reporting sedentary lifestyle (56.3 %). The chronic stressed cohort presented higher subcutaneous abdominal fat content (285 vs 319 cm^2^), and carotid intima media thickness (0.63 vs 0.66 mm; p < 0.01 for both). However, after adjustment for lifestyle/social covariates (Model 1) and biological mediators (Model 2), chronic self-perceived stress was independently associated with obesity in men (OR 2.85, 95 % CI 1.51 – 5.40) and carotid atherosclerosis in women (OR 2.262, 95 % CI 1.47 – 4.67; p < 0.01 for both).

**Conclusion:**

Our study suggests that self-reported chronic stress is an independent risk factor for obesity in men. In addition, carotid atherosclerosis was also found to be an independent risk factor in women in a Mexican population sample.

## Background

The increasing obesity prevalence is a major public health problem and it is associated with chronic health conditions such as coronary heart disease, type 2 diabetes mellitus (T2DM), and metabolic syndrome (MS) [1]. The etiology of obesity is multifaceted, involving interactions between individuals and their social, cultural, and physical environments. Psychosocial stress has also been considered as a potential factor for obesity, leading to a growing pool of clinical and epidemiological data [2]. In a number of publications, several psychosocial factors such as depression, anxiety, chronic stress, hopelessness, and certain behavioral patterns, have been linked to the development of metabolic syndrome and atherosclerosis [3]. A recent meta-analysis of longitudinal studies confirmed that psychological stress is positively related to the development of adiposity in prospective studies, although the effects were modest [4]. Stress is an ubiquitous term with no universally accepted definition. For scientific purposes, Gold and McEwen in 1998 defined “stress” as a state of threatened homeostasis; and “chronic stress” as the long-term repeated disruption of the homeostasis system. The human stressors are generally complex life events such as adversity in a relationship, overall health, work, and personal finances [5].

A growing body of literature has now provided convincing evidence that the underlying mechanisms of pathophysiological stress include inflammatory and immune processes, alterations in activating hypothalamic pituitary adrenal axis, and alterations in autonomic nervous system activity, among others. The impact of environmental factors, such as increased life stress and endogenous depression has also been linked to pathophysiological stress [5]. Cortisol hyper-secretion has been associated with a variety of diseases such as hypertension, osteoporosis, depression, and metabolic syndrome components, which include visceral obesity, dyslipidemia, and insulin resistance [6]. Such diseases have also been considered as risk factors for cardiovascular morbidity and mortality derived from atherosclerosis disease in adult populations [7]. Obesity, as the principal etiology of these metabolic disorders, has also been associated with frequent and/or persistent stimulation of the HPA axis and the accumulation of visceral fat, as a result of excessive corticosteroid [8]; thus, highlighting the importance of visceral and subcutaneous abdominal fat as a pathological mechanism of atherosclerosis disease. Psychological stress has also been considered an important risk factor for cardiovascular disease, in addition to stroke or myocardial infarction [9]. Epidemiological studies, like the INTERHEART Study [10], have used psychological stress questions, to assess subjective and perceived stressors to determine the prevalence and the impact of psychological stress. It has previously been found that chronic stress, but not periodic stress as assessed by this measure, can predict myocardial infarction and overall stroke in prospective studies [11]. Since then, several population-based studies have shown an association between psychological stress and increased risk of obesity and cardiovascular disease; however, risk estimates vary among studies, which could be due to differences in study design, ethnicity, sample size, age and gender, and different self-perceived stress measurements. Therefore, the purpose of the present study was to investigate the association of the frequency of self-perceived psychological stress with obesity, metabolic syndrome, and subclinical atherosclerosis disease in a Mexican population sample.

## Methods

### Subjects

The Genetics of Atherosclerosis Disease [Genetica de la Enfermedad Aterosclerosa] (GEA) Study was designed to investigate genetic risk factors associated with premature coronary heart disease, subclinical atherosclerosis, and other coronary risk factors in the Mexican mestizo population. A total of 2740 subjects, 1500 control subjects and 1240 coronary patients, aged 30–75 years took part in the study. The participants were not related and self-reported Mexican mestizo ancestry. All control subjects were apparently healthy asymptomatic individuals, without family history of premature coronary artery disease (CAD), recruited from blood bank donors and through brochures posted in social services centers. The coronary patient group was recruited from the National Institute of Cardiology “Ignacio Chavez” (INCICH) using their medical history. Coronary patients and control subjects with a history of renal, liver, thyroid, or malignant disease, as well as those undergoing treatment with corticosteroids, were excluded [12]. For the purpose of the present study, only the control group was included. The GEA study was approved by the Institutional Review Board of the National Institute of Cardiology “Ignacio Chavez” and the Ethics Committee of the National Institute of Genomic Medicine (INMEGEN). The study was conducted according to the Declaration of Helsinki. Written informed consent was obtained from all participants. We excluded 257 subjects with unavailable responses of the self-perceived psychological stress item. The final study population for this analysis comprised 1243 subjects. In order to determine population differences between the included participants and those excluded with no self-perceived psychological stress responses, we compared the sociodemographic and biological parameters. Statistical analyses indicated that patients in the excluded group were younger (54.2 vs 49.2 years old; p < 0.01), and no other differences were observed**.**

### Data collection

All participants answered a standardized and validated questionnaire to obtain information on family and medical history, cardiovascular risk factors, alcohol intake and smoking history, dietary habits [13], and physical activity [14]. The questionnaire applied to the entire population included a single-item question that has been used in epidemiological studies since 1970 [9–11], in order to evaluate the prevalence of self-perceived psychological stress in the studied population. In this question, stress is described as feeling tense, irritable, anxious, or as having sleep difficulties as a result of circumstances at home or at work. Participants were asked to report how often they had experienced stress, using the following incremental or graded response options: 1) Never, 2) At some point, 3) at some point during the last 5 years, 4) at several periods during the last 5 years, 5) Chronic stress during the last year, or 6) Chronic stress during the last 5 years.

### Anthropometric measures and risk factors definition

Trained personnel measured anthropometric parameters, these included waist circumference (WC) and body mass index (BMI) - calculated as weight in kilograms divided by the square of height in meters. Blood pressure was measured three times by sphygnomannometry and the last two measurements were averaged. Obesity was defined as BMI ≥ 30 kg/m^2^. Hypoalphalipoproteinemia, hypertriglyceridemia and metabolic syndrome were defined using the Adult Treatment Panel III criteria [15], except for the cut point of waist circumference [16]. Hypercholesterolemia was defined as total cholesterol levels ≥ 200 mg/dl. Hypertension was defined as systolic blood pressure ≥ 140 mmHg and/or diastolic blood pressure ≥ 90 mmHg, or the use of oral antihypertensive therapy; T2DM was diagnosed according to World Health Organization criteria [17]. Smoking history was coded as *current* versus *never* or *former* (smoking cessation at least one month before inclusion in the study); alcohol intake was defined as consumption in the last 6 months (at least one time per month) of any type of cocktail; sedentary lifestyle was defined by an item that considered whether the majority of the participant’s free time was spent reading, watching TV or resting, and not practicing any physical activity. Chronic, but not periodic stress, as assessed by the single-item questionnaire, has been previously found to be associated with cardiovascular risk [9], crude OR was calculated between obesity and the different responses of the self-perceived psychological stress item, and only the score 6 (chronic stress during the last 5 years) showed significant association to an increased risk of obesity.

### Biochemical measures

Venous blood samples were obtained after a 12-hour fasting period and all measurements were performed at the Laboratory of Endocrinology located at the INCICH, using standardized procedures. Accuracy and precision of lipid measurements in our laboratory are under periodic monitoring by the Centers for Disease Control and Prevention (Atlanta, GA, USA). Plasma glucose, total cholesterol (TC), triglyceride (TG), and High-density lipoprotein cholesterol (HDL-C) levels were measured with commercially available standardized methods. Low-density lipoprotein cholesterol (LDL-C) concentrations were estimated using Friedewald’s formula modified by De Long [18]. Homeostasis model assessment of insulin resistance (HOMA-IR) was calculated from fasting glucose and insulin measures [19].

### Tomography measures

Computed tomography of the chest and abdomen were performed using a 64-channel multi-detector helical computed tomography system (Somatom Sensation, Siemens) and interpreted by experienced radiologists. Scans were read to assess and quantify the following: 1) coronary artery calcification (CAC) score using the Agatston method [20]; 2) total abdominal, subcutaneous, and visceral adipose tissue areas, as described by Kvist [21], in order to calculate visceral adipose tissue/subcutaneous adipose tissue ratio (VAT/SAT); and 3) hepatic to splenic attenuation ratio (LSAR) as described by Longo et al. [22].

### Carotid artery intima media thickness

Carotid intima-media-thickness measurements were performed using a MicroMaxx ultrasound device (SonoSite, Inc., Bothell, WA, USA), equipped with a 7.0-14 MHz linear-array wideband transducer to obtain B-mode ultrasound images [23]. The common carotid artery, bulb and internal carotid artery were bilaterally scanned over a length of 10 mm. The mean combined outcome of the six segments was reported. The intra- and inter-observer correlation for IMT and total area using this method were 0.98 and 0.96 respectively. The cut points of IMT in Hispanic population were taken from the Ase Consensus Statement of the Society for Vascular Medicine [23].

### Statistical analyses

All analyses were performed using SPSS version 18.0 (SPSS, Chicago, II) statistical package. The self-perceived psychological stress variable was dichotomized by the subjects who reported chronic self-perceived psychological stress for >5 years in one category (Score 6), and scores 1 thru 5 composed the reference category. Data are expressed as median (interquartile range) or as frequencies for categorical variables. Comparisons were made using the t-test or the Mann–Whitney U test, and by Chi square analysis for categorical variables. Logistic regression was used to determine the independent association between chronic self-perceived psychological stress with metabolic syndrome, parameters of obesity, and subclinical atherosclerosis. Stepwise adjustments were made, starting with unmodifiable and lifestyle/social risk factors (Model 1): Age, current smoking status, alcohol intake, physical activity, and marital status. Thereafter, further adjustments for known mediating biological risk factors (Systolic BP, LDL-C, HDL-C, TG and glucose) (Model 2) were added. The values of p <0.05 were considered statistically significant.

## Results

A total of 1243 subjects, mean age 54.2 ± 9.0 years (67.2 % female), were included in the analysis. We found that 10.13 % of the subjects reported chronic stress related to circumstances at work or home during the past 5 years. Demographic characteristics and prevalence of cardiovascular risk factors according to the dichotomized categories of self-perceived psychological stress are shown in Table 1. Compared with the no chronic stress participants, the chronic stress subjects had a significantly higher prevalence of sedentary lifestyle, obesity, and increased IMT (56.3 %, 48.4 % and 37.5 % respectively). We also found a higher proportion of females in the chronic stress participants, compared to the non-chronic stress group. The anthropometric, biochemical, and tomographic characteristics of the groups are given in Table 1.

**Table 1:** Baseline, anthropometric, biochemical and tomographic characteristics by categories of self-perceived psychological stress

Risk factors	Never/periods	Chronic ≥ 5y	P value
	n = 1117	n = 126	
Female n (%)	555 (49.7)	79 (62.7)	0.006
Current smokers n (%)	242 (21.7)	30 (23.8)	0.581
Alcohol intake n (%)	119 (10.7)	12 (9.5)	0.805
Sedentary lifestyle n (%)	498 (44.6)	71 (56.3)	0.012
Hypertension n (%)	222 (19.9)	21 (16.7)	0.389
Diabetes n (%)	172 (15.4)	16 (12.7)	0.423
Dyslipidemia n (%)	183 (16.4)	24 (19)	0.422
Metabolic syndrome n (%)	480 (43)	61 (48.4)	0.243
Physical Inactivity n (%)	500 (44.8)	72 (57.1)	0.008
Overweight n (%)	538 (48.2)	48 (38.1)	0.024
Obesity n (%)	354 (31.7)	61 (48.4)	0.001
Increased IMT n (%)	170 (22)	42 (37.5)	<0.001
*Anthropometric and Biochemical*			
Age (years)	54.2 (53.7-54.8)	53.4 (52.0-54.9)	0.417
Waist circumference (cm)	95.5 (87.7-102.5)	96 (89–106.9)	0.214
BMI (kg/m^2^)	28 (25.5-31.1)	29.8 (26.4-32.3)	0.002
Systolic BP (mmHg)	114 (105 – 126)	114 (102 – 123)	0.353
TC (mg/dl)	191.8 (166–215)	190 (166.6-208.9)	0.725
LDL-C (mg/dl)	117(95.6 – 137.7)	114 (97 – 137)	0.571
HDL-C (mg/dl)	44 (36.3-54.3)	42.6 (35.1 -51)	0.309
TG (mg/dl)	149 (113–205)	152 (109–202)	0.712
Glucose (mg/dl)	91 (85 – 100)	91 (83 – 102)	0.684
HOMA-IR	4.1 (2.7-6)	4.2 (2.9-6)	0.628
Insulin (μU/ml)	17.4 (12.7-24.2)	18.2 (14–23.1)	0.510
C-reactive protein (mg/dl)	1.62 (0.82-3.36)	1.42 (0.87-3.37)	0.877
ALT (IU/L)	24 (18–34)	26 (19–35)	0.106
AST (IU/L)	25 (21–31)	25 (22–32)	0.120
*Tomographic and ultrasonography*			
TAF (cm^2^)	443 (359–547)	480 (377–583)	0.035
SAF (cm^2^)	285 (218–366)	319 (242–409)	0.008
VAF (cm^2^)	153 (115–200)	156 (106–192)	0.706
IMTave (mm)	0.63 (0.55-0.73)	0.66 (0.60-0.83)	<0.001

Differences between groups with the Chi square test for proportions and U-Mann Whitney test for median (range). IMT = Intima media thickness. BMI = Body mass index, BP = Blood pressure, TC = Total cholesterol, LDL = Low density lipoprotein, HDL = High-density lipoprotein, TG = Triglycerides, HOMA-IR = Homeostasis model assessment of insulin resistance, ALT = Alanine transaminase, AST = Aspartate transaminase, TAF = Total abdominal fat, SAF = Subcutaneous abdominal fat, VAF = Visceral abdominal fat and IMTave = Intima media thickness average

The subjects with chronic self-perceived stress showed higher BMI, total abdominal and subcutaneous abdominal fat, and carotid IMT average when compared with their counterparts. No significant differences were observed in the biochemical characteristics, including lipid profile, insulin, HOMA-IR, hepatic enzymes, and C-reactive protein. When all these characteristics were analyzed by gender (data not shown), chronic-stressed men were younger and had elevated systolic BP compared to those without stress; also chronic-stressed women presented lower HDL-C, and higher AST levels compared to those without stress. Moreover, chronic-stressed women showed the greatest carotid intima media thickness.

Logistic regression models were used to assess whether self-perceived chronic stress was associated with metabolic syndrome or its individual components, obesity, and carotid atherosclerosis (Table 2). Compared with non-chronic stress females, the chronic stress female cohort were more likely to have hypoalphalipoproteinemia, independent of age, current smoking status, alcohol intake, physical activity, and marital status (Model 1). However, after full adjustment the association was no longer independent. The chronic stress women showed an independent association with increased IMT (Model 2), this association not only remained significant after full adjustment for confounding factors, the association even increased. In the chronic stress men cohort, there were no significant associations for the metabolic syndrome and its individual components. However, we found a significant association with obesity in both models.

**Table 2:** Odds ratio (OR) of chronic self-perceived psychological stress for metabolic risk factors and subclinical atherosclerosis by gender

Metabolic risk factors	Female n = 79		Male n = 47	
	OR (95 % CI) P value	OR (95 % CI) P value	OR (95 % CI) P value	OR (95 % CI) P value
	Model 1	Model 2	Model 1	Model 2
Systemic hypertension	0.66 (0.32 – 1.37) 0.27	0.59 (0.28 – 1.26) 0.17	1.12 (0.52 – 2.40) 0.75	0.95 (0.43 – 2.06) 0.90
Hyper trigliceridemia	1.31 (0.81 – 2.12) 0.27	0.61 (0.29 – 1.29) 0.20	0.89 (0.48 – 1.66) 0.72	0.94 (0.43 – 2.05) 0.88
Hypoalpha lipoproteinemia	2.13 (1.26 – 3.50) 0.004	1.33 (0.80 – 2.20) 0.26	0.92 (0.50 – 1.71) 0.81	0.71 (0.37 – 1.36) 0.30
Diabetes Mellitus	0.81 (0.38 – 1.71) 0.58	0.74 (0.34 – 1.60) 0.45	0.98 (0.41 – 2.32) 0.96	0.74 (0.28 – 1.92) 0.74
Metabolic Syndrome	1.46 (0.89 – 2.37) 0.12	1.45 (0.75 – 2.79) 0.26	1.05 (0.57 – 1.94) 0.85	1.12 (0.45 – 2.78) 0.79
Obesity	1.52 (0.92 – 2.51) 0.09	1.44 (0.86 – 2.41) 0.15	2.89 (1.55 – 5.30) 0.001	2.85 (1.51 – 5.40) 0.001
SAF p > 75	1.49 (0.91 – 2.43) 0.11	1.81 (0.88 – 3.71) 0.10	1.50 (0.77 – 2.92) 0.22	0.46 (0.16 – 1.30) 0.14
VAF p > 75	1.29 (0.77 – 2.15) 0.37	1.15 (0.60 – 2.22) 0.66	1.62 (0.83 – 3.16) 0.15	1.14 (0.48 – 2.67) 0.75
TAF p > 75	1.19 (0.72 – 1.96) 0.49	0.93 (0.40 – 2.17) 0.88	1.30 (0.67 – 2.51) 0.43	0.38 (0.13 – 1.14) 0.08
Increased IMT	2.38 (1.35 – 4.10) 0.003	2.62 (1.47 – 4.60) 0.001	0.81 (0.32 – 2.04) 0.66	0.78 (0.30 – 1.99) 0.60

All associations were tested using logistic regression

Adjustment model = M 1 = age, current smoking, alcohol intake, physical activity, marital status

Adjustment model = M 2 = Model 1 + Systolic BP, HDL-C, LDL-C, TG and Glucose

Hipertriglyceridemia = TG >150 mg/dl, Hipoalphalipoproteinemia = HDL <40 mg/dl in men and < 50 mg/dl in women, SAF = Subcutaneous abdominal fat, VAF = Visceral abdominal fat, TAF = total abdominal fat and IMT = Intima media thickness

## Discussion

Our findings showed an independent association between chronic self-perceived stress (measured by a single-item questionnaire) and obesity, in males; and carotid atherosclerosis in female patients, which was previously reported to be linked to ischemic stroke [9].

We also showed an association between chronic stress and carotid atherosclerosis in women. Chronic hypersecretion of stress mediators such as cortisol, in individuals with genetic predisposition exposed to a permissive environment [24], is an established mechanism to the development of hypertension, dyslipidemia, type 2 diabetes mellitus, and atherosclerosis disease [25, 26]. Our results are supported by a large population sample study that investigated the association between psychological stress and carotid atherosclerosis. Our findings indicated that high psychological stress and carotid plaques were independently and linearly related, with plaque prevalence odds of 1.55 (95 % CI 1.10 to 2.18, p = 0.011) among women and 1.38 (95 % CI 1.12 to 1.75, p = 0.005) among men [27].

In this study, an independent association to chronic self-perceived stress was only found for sedentary lifestyle and BMI, as cardiovascular risk factors in the Mexican population. However, another study that investigated the relationship between stress at work and cardiovascular indicators (such as blood pressure and cardiovascular symptoms) using the Karasek’s model in a Mexican cohort (n = 109 nurses), found a significant association between job insecurity and cardiovascular indicators; pointing out the importance of these variables and their effects on stress in the Mexican work setting [28].

In the Malmo Preventive Project, a prospective study of 13,609 (2741 women) participants, self-reported chronic stress significantly increased the risk of suffering cardiovascular disease like ischemic heart disease or stroke events in middle-aged men. For women, the trends were similar but not significant after adjustment for unmodifiable, social, lifestyle risk factors, and biological parameters [29]. In contrast, we found a significant greater risk of increased intima media thickness present in women who reported chronic stress, and for obesity for men. However, the samples’ characteristics and size were too small to investigate causality.

The underlying mechanisms by which different forms of mental stress increase the risk of obesity, particularly the abdominal phenotype, are complex. This has been ascribed to individual maladaptation to chronic stress exposure mediated by a dysregulation of related neuroendocrine axes. Alterations in the control and action of the hypothalamic-pituitary-adrenal axis may play a major role in this context, with the participation of the sympathetic and parasympathetic nervous system, systemic inflammation, or an interaction between genetic predisposition and stress. Specifically, the inability to cope with psychological stress, particularly early stress in life, ultimately leads to an increase of circulating catecholamines and cortisol, which in turn leads to loss of appetite control and increased adiposity [29], and resulting in an elevated heart rate and blood pressure, coronary constriction, accumulation of lipid in the intima, and electrical heart instability [30].

True comparisons between these studies and the one presented in this article are rather difficult for various reasons, mainly because of differences in methodology, ethnicity, cohort, and the participants’ age. Our principal strength is the robust association found between chronic stress, obesity, and carotid atherosclerosis, following adjustment for established risk factors. This suggests that stress may play an independent role in the development of cardiovascular disease in the Mexican population.

The shortcomings of the study are identified as follows. First, the cross-sectional design of the GEA study does not allow us to determine causality as it relates to the effect of permanent self-perceived psychological stress on obesity. Second, the population-based study is not a representative and randomized cohort of Mexican population; however, the prevalence of overweight and obesity are similar to that reported by ENSANUT 2012 [31]. Third, we had missing data of the stress item in the first 254 subjects, and we were only able to perform the carotid artery ultrasound in 914 subjects.

## Conclusions

In summary, we found that self-perceived chronic stress is an independent risk factor for obesity in men and carotid atherosclerosis in women, in the Mexican population. Our results suggest the need for further prospective studies in larger Hispanic populations addressing the potential role for psychological stress as a risk factor for cardiovascular disease. Confirmatory analyses of the association found especially in longitudinal settings, are also needed. Furthermore, in order to give insight as to which subcomponents of perceived stress are of importance, more elaborate measures of psychological stress are suggested.
